# Supplementary material for: Effect of dexamethasone pretreatment using deep learning on the surgical effect of patients with gastrointestinal tumors
Source: PLoS One. 2024 Jul 17;19(7):e0304359. doi: 10.1371/journal.pone.0304359 (PMC11253962; doi:10.1371/journal.pone.0304359)
Supplement: Attachment — Submitted filename: Response to Reviewers.docx [file pone.0304359.s002.docx]

In this study, the influence of AI-controlled dexamethasone administration in gastrointestinal tumor surgery on postoperative outcomes is investigated. It is encouraging to see that AI algorithms are being studied prospectively.
However, your work shows substantial weaknesses. Neither the title nor the abstract provide sufficient information on the methodology and outcome parameters. The introduction lacks a clear structure and a common thread. The study concept cannot be deduced from this.

Reply: Thank you for your suggestion. We have provided additional explanations on the abstract methods and results in this revision; Adjustments have been made to the introduction section to make it more fluent.
With regard to the methodology, it is noticeable that the study was not registered in a study register. However, I also cannot understand the study population or the group classification. How do the groups differ? Frailty is one of the main risk factors for the development of delirium and POCD and was not recorded. It is not known to what extent regional anesthesia or epidural anesthesia was used. With regard to cognitive evaluation, non-standardized methods were used.

Reply: Thank you for your suggestion. There was an error in the description of the grouping situation, and adjustments have been made to explore the impact of preoperative application of artificial intelligence-controlled dexamethasone anesthesia on patients. Therefore, the control group is a blank group, forming a significant contrast with the experimental group; This study did not involve any risk factors, therefore there are no relevant records. This study was preoperative anesthesia, using inhalation and intravenous anesthesia, as described in section 2.3. Epidural anesthesia was not involved, and the scope of anesthesia was general anesthesia. In this modification, it has been supplemented; Regarding cognitive evaluation, this revision has added the Montreal Cognitive Assessment Scale (MoCA) and related results.
The low standard deviation of the results is striking in the context of the group size. Very surprising is the result with a halving of the POCD by an AI-controlled administration of 8mg dexamethasone, which in this patient population speaks for an insufficient allocation to the groups, since in the context of such a disease, intervention and co-morbidities with multiple influencing factors by such an intervention does not seem very likely.

Reply: Thank you for your suggestion. There was an error in the description of patient allocation. In order to explore the impact of preoperative application of artificial intelligence-controlled dexamethasone anesthesia on patients, the control group was a blank control group, compared with the experimental group dexamethasone group. Therefore, there was a difference in postoperative POCD in patients. Previous research results may not have a unified view on this point. This study showed that preoperative application of artificial intelligence-controlled dexamethasone anesthesia was beneficial for reducing postoperative POCD in patients. In this modification, we have rephrased the grouping and handling of patients, and adopted a new method to evaluate their cognition.
