# Supplementary material for: Effect of dexamethasone pretreatment using deep learning on the surgical effect of patients with gastrointestinal tumors
Source: PLoS One. 2024 Jul 17;19(7):e0304359. doi: 10.1371/journal.pone.0304359 (PMC11253962; doi:10.1371/journal.pone.0304359)
Supplement: Attachment — Submitted filename: Response to Reviewers.docx [file pone.0304359.s004.docx]

Reviewer #1: thanks
the revised paper is better than the original one
i recommends adding recent references (in the last 5 years)

Reply: Thank you for your suggestion. I have reviewed the literature and ensured that the most recent references are added where applicable to keep our content up-to-date with the latest research findings and developments.

your hard work is clear
for my point of view the paper now is suitable for publication.
congratulations

Reviewer #2: I have reviewed the revised submission. After going through it, I still seem to require more clarifications.
1. Please clarify two different statements written about exp and ctrl groups:
In Abstract: The participants were randomly assigned to either the control (Ctrl) group (n=40) or the experimental (Exp) group (n=40). The Ctrl group received a 2 mL intravenous injection of 0.9% saline solution 1-2 hours prior to surgical incision, while
the Exp group received a 10 mg (2 mL) intravenous injection of dexamethasone at the same time point.
While in Research Object heading: Participants were randomly assigned to two groups based on the preoperative anesthesia pretreatment methods implemented. In the control (Ctrl) group, patients received an intravenous injection of 10 mg (2 mL)
dexamethasone administered manually 1-2 hours prior to skin incision. Conversely, the experimental
(Exp) group underwent anesthesia detection utilizing deep learning technology in addition to the
anesthesia pretreatment protocol administered to the Ctrl group. these seem to be opposing statements.

Reply: Thank you for providing the modification suggestions. We understand your concerns and appreciate your feedback.

The inconsistency between the description of the experimental group and the control group in the abstract and research subject title is our mistake. Here, we clarify as follows:

In our study, we did have two groups: the control group (Ctrl) and the experimental group (Exp). The description mentioned in the title of the research subjects is correct, that is, the control group received dexamethasone injection, while the experimental group received dexamethasone injection assisted by a deep learning-based anesthesia detection system.

However, the description in the abstract is inaccurate. We deeply apologize for this. The correct description should be: participants were randomly assigned to a control group and an experimental group. In the control group, patients manually received intravenous injection of dexamethasone 1-2 hours before the skin incision. On the contrary, the experimental group used deep learning techniques to monitor anesthesia status on the basis of the control group.

We thank you again for pointing out this issue and sincerely apologize for any inconvenience caused by this confusion. We have revised the title to reflect the true situation of the experiment.

2. Please clarify the use of Deep Learning algorithms as a research tool for this comparative study of two groups. You have mentioned that these were used in a sample of 10 patients. What was the rationale for not using in all patients?

Reply: Thank you for providing the modification suggestions. Regarding the application of deep learning algorithms in anesthesia detection systems evaluated using 10 patients in the study, no comparison was made between the two groups of patients. Its main purpose was to test the performance of the deep learning-based BIS detection system constructed in the study. The reason why it was not used in all patients is because deep learning algorithms usually require a large amount of computing resources and time for training and analysis. In some cases, we may not have sufficient computing resources to process data for the entire patient population. Therefore, to avoid resource waste, we choose to use these algorithms in smaller samples.

3. To me as a clinician the study seems to compare the beneficial effects of pre-anesthetic use of dexamethasone on post-operative cognitive behaviour and Gastrointestinal recovery of patients. Dexamethasone affects teh neuronal networks of the body, part of our deep learning networks. Actual tests performed in both groups were the use of MoCA, GI functional tests and other lab tests. The table 1 based on Deep Learning is given for a sample of 10 patients. My query is: even if you remove the table 1and do not employ deep learning algorithms in this study, how would it affect your results? Your data is still showing the beneficial effectg of using dexamethasone. Please clarify in your introduction and methodology how was this technology used and what difference was noted in two groups.
In Conclusion: You have mentioned the clear benefits of using Deep Learning processing on post-operative recovery of patients. It seems this statement has been used as synonym for use of dexamethasone as there is no mention of the medication in concluding statements. Please clarify it, too.
In summary, please make your manuscript as elaborate, as possible, for clinicians and physicians to understand clearly.

Reply: Thank you very much for reviewing our research and providing valuable suggestions for revisions. Here is my response to your question and corresponding modifications:

(1) Based on the above modifications, the control group was given dexamethasone, while the experimental group was given dexamethasone with the assistance of a deep learning-based anesthesia detection system. Therefore, if Table 1 is deleted and the deep learning algorithm is not used for grouping, it will not form a control analysis. In addition, in the introduction and methodology, we will provide a detailed explanation of how deep learning algorithms are applied in our research, clarify the differences between the two groups, and ensure that readers can clearly understand the technical details in the study.

(2) In the conclusion section, we will clarify that the benefits of deep learning processing are not synonymous with the use of dexamethasone. We will clarify this point and ensure that the conclusion section clearly expresses the importance of dexamethasone in postoperative recovery of patients, while not confusing the benefits of deep learning processing with the effectiveness of drug therapy.

We will write the manuscript as detailed as possible according to your suggestions to ensure that clinical doctors and doctors can clearly understand our research. Thank you again for your suggestions and review.
